# QuickStats

**Published:** 2015-02-13

**Authors:** FROM THE NATIONAL CENTER FOR HEALTH STATISTICS

**Figure:**
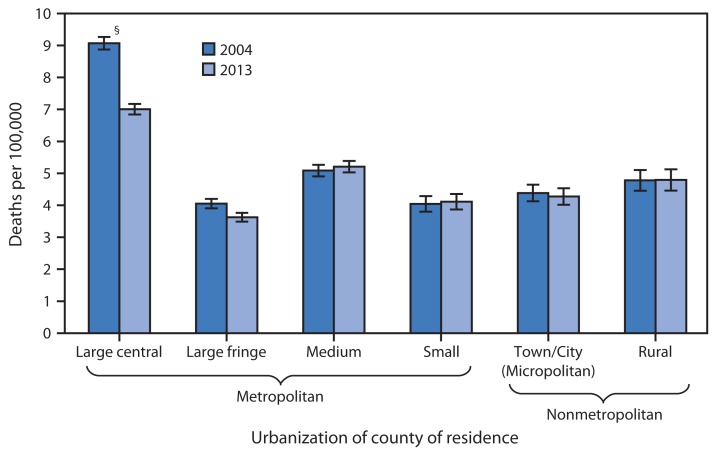
Age-Adjusted Homicide Rates,* by Urbanization of County of Residence^†^ — United States, 2004 and 2013 *Age-adjusted rates per 100,000, based on the 2000 U.S. standard population. Deaths from homicide are coded *U01–*U02, X85–Y09, and Y87.1 in the *International Classification of Diseases, 10th Revision*. ^†^Counties were classified into urbanization levels based on a classification scheme that considers metropolitan/ nonmetropolitan status, population, and other factors. ^§^95% confidence interval.

From 2004 to 2013 in the United States, the age-adjusted homicide rate in large central metropolitan counties decreased 23% (from 9.1 to 7.0 deaths per 100,000 population), and the rate in large fringe metropolitan counties (suburbs of large cities) decreased by 10% (from 4.1 to 3.6). For four other county urbanization types (medium and small metropolitan and town/city [micropolitan] and rural nonmetropolitan), rates in 2004 and 2013 were similar. For both years, the homicide rates in large central metropolitan counties were higher than the rates for all other county types, and the rates for medium metropolitan counties were higher than the rates for large fringe and small metropolitan counties, and town/city (micropolitan) nonmetropolitan counties. Overall, in the United States, the 2004 age-adjusted homicide rate was 5.9 deaths per 100,000 population, and the 2013 rate was 5.2.

**Source:** National Vital Statistics System. Available at http://wonder.cdc.gov

Ingram DD, Franco SJ. NCHS urban-rural classification scheme for counties. National Center for Health Statistics. Vital Health Stat 2 2012(154).

**Reported by:** Deborah D. Ingram, PhD, ddingram@cdc.gov, 301-458-4733; Li-Hui Chen, PhD.

